# Succinate Dehydrogenase Complex Iron Sulfur Subunit B (SDHB) Immunohistochemistry in Pheochromocytoma, Head and Neck Paraganglioma, Thoraco-Abdomino-Pelvic Paragangliomas: Is It a Good Idea to Use in Routine Work?

**DOI:** 10.31557/APJCP.2021.22.6.1721

**Published:** 2021-06

**Authors:** Aylin Ege Gul, Sevinc Hallac Keser, Nagehan Ozdemir Barisik, Yesim Saliha Gurbuz, Sibel Sensu, Nusret Erdogan

**Affiliations:** 1 *Health Sciences University, Kartal Lutfi Kirdar Research&Traning Hospital, Pathology Clinic, Istanbul, Turkey. *; 2 *Pathology, Istınye University Medical Faculty, Turkey.*

**Keywords:** Pheochromocytoma, paraganglioma, SDHB, immunohistochemistry

## Abstract

**Background:**

In this study, we aimed to detect Succinate Dehydrogenase Complex Iron Sulfur Subunit B (SDHB) frequency in paragangliomas and pheochromocytomas (PPGL) with immunohistochemistry; compare with Pheochromacytoma of the Adrenal Gland Scaled Score (PASS) classification and analyse the differences between pheochromocytoma (Pheo), head-neck paragangliomas (HNPGL) and thoraco-abdominal-pelvic paraganglioma (TAPPGL) sub-groups.

**Methods:**

A total 114 PPGL cases (73 HNPGL, 15 TAPPGL and 27 Pheo belonging to 112 cases) are included. Immunohistochemically, SDHB and Ki-67 are investigated and malignancy risks are determined by PASS classification. Results are assessed statistically with chi-square test and p <0,01 is considered significant.

**Results:**

*SDHB* mutations are observed in 20 of 114 (17.54 %) PPGL cases, 3 (11,12%) of which is Pheo, 12 (16,44) is HNPGL, and 5 (35,71%) is TAPPGL (P <0,02). While 15/82 (18,29%) cases with *SDHB* mutations do not have a malignancy potential according to PASS classification, 5/32 (15,63%) cases has (p=0,73). TAPPGL, HNPGL and Pheo sub-groups have a significant difference between SDHB expression (p <0,02), malignancy potential according to PASS classification (p <0,0001) and Ki-67 proliferation index (p <0,0001).

**Conclusion:**

To identify patients for molecular pathological examination, routine application of SDHB immunohistochemistry to PPGL tumors are suggested especially in HNPGLs.

## Introduction

Paragangliomas and pheochromocytomas (PPGLs) are neuroendocrine tumors derived from the parasympathetic or sympathetic nervous system. Pheochromocytomas (Pheo) arise from the adrenal medulla and paragangliomas (PGLs) from the extra-adrenal paraganglia. PGLs arising from head and neck (HNPGL) are usually derived from parasympathetic paraganglia and are non-functional. On the contrary, thoraco-abdominal-pelvic paragangliomas (TAPPGL) are usually derived from the sympathetic ganglia, as well as Pheo (McKenny, 2018; Donato et al., 2019). Actually, the only marker for malignancy is the existence of metastases. Yet, malignant Pheos are usually larger with more necrotic areas, and ratio of smaller cells is higher than benign Pheos (McKenney, 2018).

PPGLs might be sporadic or familial and genetic mutations are detected in almost 40% of PPGLs (Donato et al., 2019; Plouin et al., 2016; Benn, 2015). However, patients with hereditary paraganglioma-pheochromocytoma syndrome might reveal no family history of paraganglioma or pheochromocytoma and might present with a single tumor located at skull base, neck, thorax, abdomen, adrenal, or pelvis (Neumann et al., 2008). In familial and some sporadic cases, *PGL/Pheo* mutations in succinate dehydrogenase complex (SDHx) have been identified. Succinate dehydrogenase (*SDH*) mutations are mitochondrial DNA mutations. They have been described in some other types of cancer; they change mitochondrial metabolism, increase risk of tumorigenesis and allow cancer cell modification to changing environments (Nazar E, Khatami F, Saffar H, Tavangar SM, 2019). Fumaratehydratase (FH) and SDH (SDHB, -C and -D) are mitochondrial Krebs cycle enzymes which act as tumor suppressors (Nazar et al., 2019; Pollard et al., 2005). Although mutations in all subunits occur in cancer, tumors containing mutations in the catalytic subunit SDHB are predominantly malignant and associated with enhanced risk of metastasis (Nazar et al, 2019; Yang et al., 2013). Germline mutations in SDHx are responsible for up to 30% of the cases (Donato et al, 2019; Plouin et al., 2016; Kantorovich et al, 2010; Baysal , 2003; Gunawarde et al., 2017). Loss of SDHB immune expression is a marker for the mutation in one of the five *SDH* genes. Recognition of hereditary Pheo cases is crucial for identifying mutation-positive patients and their families (Nazar et al., 2019; Khatami et al. 2018; Castelblanco et al., 2013; Bryant et al., 2003; Albattal et al., 2019; Maria Curras-Freixes et al, 2015).

Our general objective was to immunohistochemically detect *SDHB*-mutation frequency in PPGL tumors, to compare with PASS classification and to analyse the differences between Pheo, HNPGL and TAPPGL 

## Materials and Methods

*Cases*

A total 114 PPGL tumors (73 HNPGL, 14 TAPPGL and 27 Pheo) belonging to 112 cases diagnosed between March 2007 and January 2020 were included in this single-centered retrospective study. Data on age and sex of patients, location and size of the tumors, metastasis and survival status were retrieved from Pathology Archive. Survival data was obtained from death reporting system which was a part of the hospital information management system. 

*HIstopathological Evaluation*

Hematoxylin-eosin stained slides of the study were re-evaluated by two independent pathologists regarding the presence of a diffuse growing pattern, necrosis, high cellularity, monotonous structure, cell spindling, mitosis in 10 high power field (HPF), atypical mitosis, capsular invasion, peripheral adipose tissue invasion, vascular invasion, marked nuclear pleomorphism, and hyperchromasia. Pheochromacytoma of the Adrenal Gland Scaled Score (PASS) system was used to score all tumors according histopathological features. The malignancy potential of each tumor was detected according to PASS classification and score> 3 was accepted as a tumor with malignant potential (Table 1).

*Immunohistochemical Staining*

In all cases, a representative paraffin block that contains both tumor and normal tissue was chosen for immunohistochemical *SDHB* mutation analysis and Ki-67 labeling index. Immunohistochemical examination was performed on deparaffinized sections using the standard avidin–biotin–peroxidase complex method with automated immunostainer (BenchMark ULTRA, Ventana medical system, Tucson, AZ, USA). Formalin-fixed paraffin-embedded blocks were analyzed for expression of rabbit monoclonal antibody SDHB (Epitomics an Abcam, clone:EP288, dilüsyon:1/20, Burlingame, CA,USA). Cytoplasmic SDHB staining was lost in cases with mutations. If SDHB staining in the tumor cells was evidently less intense compared to non-tumoral cells, or if it showed a weak diffuse cytoplasmic blush instead of a granular staining pattern, SDHB immunohistochemistry was considered, negative. Intratumoral vascular structures and peripheral non-tumoral tissues were used as positive control. 

Ki-67 antibody (Thermoscientific, clone: SP6, dilüsyon:1/150, Kalamazoo, Michigan, USA) was used to identify proliferative activity. The regions with highest concentrations of Ki-67 positive nuclei were selected and evaluated with a high-power magnification (×400) in tumors. On the basis of 1000 neoplastic nuclei, Ki-67 labeling ndex was calculated in each slide as the percentage of immunopositive nuclei. The cases were classified in two categories according to immunoreactivity ≤3% and >3%. 

*Statistical Analysis*

All statistical analyses were carried out using Stata 16 (Stata Corp LP, Texas, USA). Characteristics of patients were evaluated with descriptive analysis. Differences between Pheo, HNPGL and TAPPGL, according to SDHB mutation, malignancy potential (PASS-based) and Ki-67 index, were analysed with chi-square group comparison test. Pearson correlation performed to document correlation between parametric variables. Ordinary logistic regression performed for binary variables relations.P≤0.05 was considered statistically significant. 

Ethical approval was obtained from Istinye University Human Studies Ethical Committe (Nr: 62/2020).

## Results

*Clinical Findings*

Seventy-four of the patients were female and 38 were male. Age of the patients were between 23 and 76 (mean age: 51.99). Nine of the patients were ≤ 30 and 103 patient were >30 years old. Mean age of Pheo was 46.8 years (24-71 years); 4 cases (14.81%) were between 0 – 30 , 23 cases (85.18%) ≥ 31years old. Mean age of HNPGL was 53.93 (23-76); 3 cases (4.10%) were 0 – 30 and 70 cases ( 95.89% ) were ≥ 31 years old. Mean age of TAPPGL was 51.71 (26-68); 2 cases (14.28%) were 0 – 30 and 12 cases (85.71%) were ≥ 31 years old (Table 2). Mean diameter of PPGL was 4.51 cm (0.4cm-18cm); 6,92 cm (2.4cm-14cm) for Pheo, 3.29 cm (0.40-8cm) for HNPGL and 6.6 cm (1.50-18cm) for TAPPGL. Twenty-seven of the tumors were located at adrenal gland and diagnosed as Pheo. The other cases were extraadrenal and considered as PGL. Seventy-three of the PGL cases were located at neck (HNPGL); 49 of them were close to carotid, 19 at neck, 5 at middle-ear. Fourteen tumors were TAPPGL; 2 paraaortic cases, 5 retroperitoneal cases, 3 lomber cases, one intraabdominal, one urinary bladder, one L4 intradural, and one pleural case were detected. In one case, two tumors were excised in the same operation, one was from right surrenal gland and the other from retroperitoneum. In another case, a tumor near to right carotid and after a short time, to left carotid were resected. 

Metastases were found in four cases: The tumor on right carotid had metastases to lung, sacrum and brain, and the patient died 11 months after brain metastasis. One of the two Pheos located on left adrenal gland metastasized to lymph node and the other, to bone. The PGL located on retroperitoneum metastasized to liver. The case with Pheo metastasized to lymph node, had also a renal cell carcinoma of right kidney.

Five cases died during follow-up (Four HNPGL and one case in urinary bladder location). PGL on right carotid had malignancy potential due to PASS and 11 months after brain metastasis died due to multiple metastases (also to lung and sacrum). The case with urinary bladder PGL died 9 months after the operation. One of the HNPGL cases died 6 days after surgery due to postoperative complications. The other two cases died at month 37 and 70. 

*Histopathological Findings*

On histopathological examination, 2 cases were diagnosed as gangliocytic PGL, one case as composite PGL and one case as composite pigmented Pheo. 

Microscopically, in 11 cases (9.65%) a diffuse growth pattern, in 11 cases (9.65.%) necrosis, in 9 cases (7.89%) high cellularity, in 8 cases (7.02%) monotonous appearance , in 22 cases (19.3) spindle cells, and in 4 cases (3.51%) mitosis > 3/ 10 HPF10 were detected. In three cases (2.63%) atypical mitosis were found. Pleomorphism in 46 (40.35%) cases, and hyperchromasia in 51 cases (44.74%) were noted. In 71 cases (62.28%) capsule, in 10 cases (8.77%) adjacent adipose tissue and in 10 cases (8.77%) vascular invasion were found.

Regarding PASS results; 16 cases (14.04%) were 0 point, 33 (28.95%) were 1, 14 (12.28%) 2, 19 (16.67%) were 3, 9 (7.89%) were 4, 6 (5.26%) were 5, 3 (2.63%) were 6, 3 (2.63%) were 7, 2 (1.75%) were 8, 5 (4.39%) were 9, 2 (1.75%) were 10, 1 (0.88%) was 14 and one was 16 points. Generally, in 32 cases (28.07 %) (14 in Pheo (%51.85), 11 in HNPGL (15.07%),7 in TAPPGL (50.00%), PASS was ≥4. These cases were considered to have a malignant biological behavior (Table 2). In all 4 metastatic cases, malignancy potential existed according to PASS classification (score≥4); but one of them was SDHB positive. 

*Immunohistochemical Findings*

In 20 (17.54%) of the tumors (3 (11.12%) of Pheo, 12 (16.44) of HNPGL, 5 (35.71%) of TAPPGL) *SDHB* mutation was detected, immunohistochemically. *SDHB *mutation was detected in 4 (40.00%) cases age under 30, and in 16 (15.38%) cases age ≥31years. One of the cases with *SDHB* mutation was 62 years old patient with 2 tumor foci, one at adrenal gland and one at retroperitoneum. One of 4 metastatic cases (25%) had *SDHB* mutation. Ki-67 index was over 3% in 29 cases (25.43%) (3 in Pheo (11.12%), 19 in HNPGL (26.03%),7 in TAPPGL (50.00%) group) (Table 2). 

*Statistical Findings*

All statistical data were summarized in Table 2, 3 and 4. There was a statistically significant difference between Pheo, HNPGL and paragangliomas in other locations according to* SDHD* mutation presence (P<0.029), malignancy potential according to PASS (over 3 or equal and lower 3) (P<0.0002) and, Ki-67 index (over 3 or equal and lower 3) (P<0.0007) (Table 2). 

**Table 1:** Pheochromocytoma of the Adrenal Gland Scale (Thompson LDR, 2020)

Microscopic feature	Score
Vascular invasion	1
Capsular invasion	1
Periadrenal adipose tissue invasion	2
Cell nests of large proportions or diffuse growth	2
Focal necrosis or confluent necrosis	2
High cellularity	2
Cellular monotony	2
Mitotic figures >3/10HPF	2
Atypical mitotic figures	2
Marked nuclear pleomorphism	2
Hyperchromasia	1

**Table 2:** SDHD Mutation, Malignancy Potential, Age and Ki-67 Index in Cases with Paraganglioma and Pheocromocytoma

		Paraganglioma		Pheochromocytoma	Total	
		N=87 (%)		N=27 (%)	N=114 (%)	P
		Others	Head/Neck			
		N=14	N=73			
SDHD mutation	(-)	9 (64.29)	61 (83.56)	24 (88.88)	94	
	(+)	5 (35.71)	12 (16.44)	3 (11.12)	20	P<0.029
Malignancy potential	PASS≤3	7 (50.00)	62 (84.93)	13 (48.15)	82	
	PASS>3	7 (50.00)	11 (15.07)	14 (51.85)	32	P<0.0002
Age	Mean±SD	51.7±14.6	53.9±13.4	46.8±14.2	51.9±13.9	
	Range	(26-68)	(23-76)	(24-71)	(23-76)	
	0 - 30	2 (14.28)	3 (4.11)	5 (18.5)	9*	
	≥31	12 (85.72)	70 (95.89)	22 (81.5)	103*	P<0.13
Ki-67	≤%3	7 (50.00)	54 (73.97)	24 (88.88)	85	P<0.0007
	>%3	7 (50.00)	19 (26.03)	3 (11.12)	29	

*, Two patients had two different types of tumors; SD, Standard deviation

**Figure 1:**
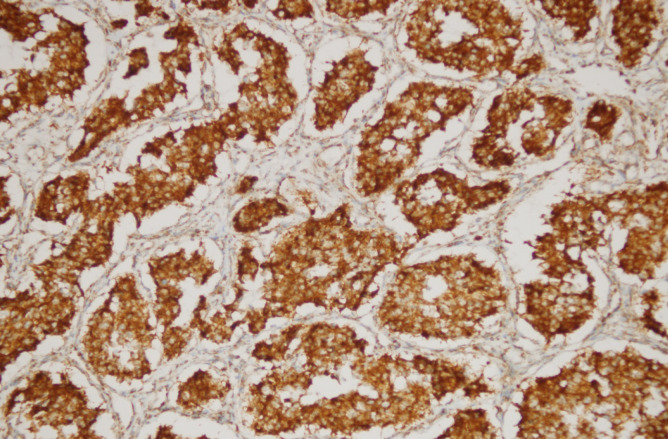
Diffuse Intracytoplasmic SDHB Immunoreactivity in a Head and Neck Paraganglioma (Immunohistochemistry SDHBX200)

**Figure 2:**
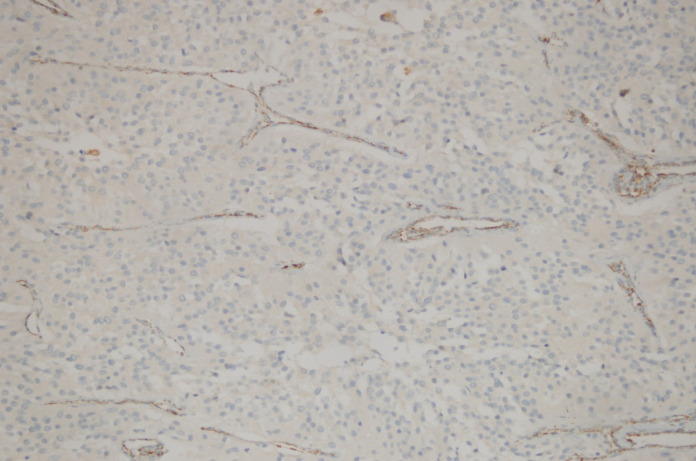
Loss of SDHB Immunoreactivity in a Thoracoabdominopelvic Paraganglioma and Positive Dotty Immunostaining in the Vessel Walls (Immunohistochemistry SDHBX200)

**Table 3:** Comparison between SDHB Mutation and Age

	Age group			P
SDHB	0 – 30	≥ 31	Total	
	N (%)	N (%)	N (%)	
(-)	6 (60)	88 (84.6)	94 (82.5)	p<0.07
(+)	4 (40)	16 (15.4)	20 (17.5)	
Total	10	104	114	

**Table 4:** Malignancy Potential of the Cases Detected According to PASS Classification

	Malignancy Potential*			P
SDHB	PASS≤3	PASS>3	Total	
	N (%)	N (%)	N (%)	
(-)	67 (81.7)	27 (84.4)	94 (82.5)	p>0.5
(+)	15 (18.3)	5 (15.6)	20 (17.5)	
Total	82	32	114	

*PASS score> 3 was accepted as a tumor with malignant potential.

**Figure 3:**
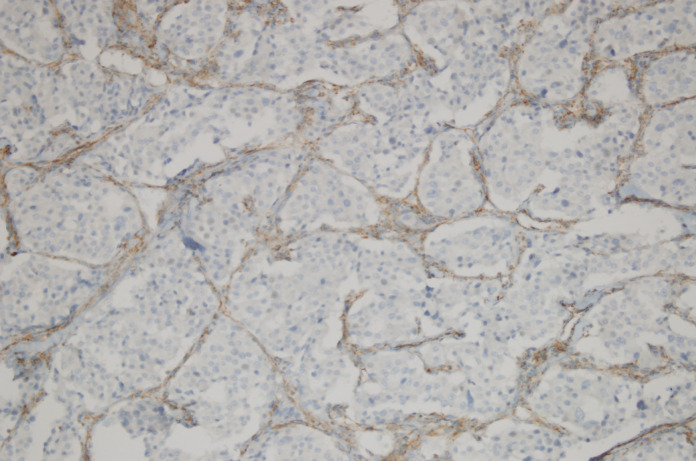
Loss of SDHB Immunoreactivity in a Pheochromoctoma and Positive Dotty Immunostaining in the Sustentacular Cells and Vessel Walls (Immunohistochemistry SDHBX200)

**Figure 4:**
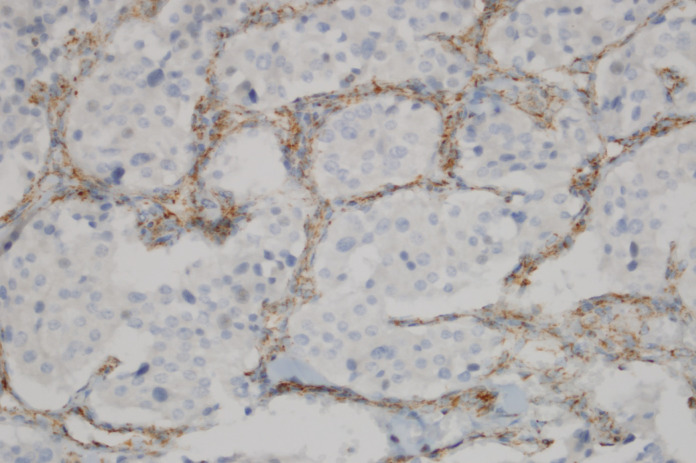
Loss of SDHB Immunoreactivity in the Chief Cells of a Pheochromoctoma and Positive Dotty Immunostaining in the Sustentacular Cells and Vessel Walls (Immunohistochemistry SDHBX400)

## Discussion

PPGL is a heterogenous group of neural crest-derived tumors. Microscopically, the tumor cells are characteristically arranged in well-defined nests (‘Zellballen’) bound by a delicate fibrovascular stroma. They vary considerably in size and shape, and have a finely granular basophilic or amphophilic cytoplasm. The nuclei are usually round or oval with prominent nucleoli and may contain inclusion-like structures resulting from deep cytoplasmic invaginations. Common features such as giant nuclei and hyperchromasia are not a feature of malignancy. Histopathological features of PPGL resemble each other, but biological behaviours of Pheo, TAPPGL and HNPGL are different. As a rule, malignant PPGLs have distant metastases, commonly found in the liver, lung, bone, and lymph nodes. The term ‘metastatic PPGLs’ has been used to replace ‘malignant PCC/PGL’ in the latest WHO endocrine tumors classification (Lam, 2017). 

Morphologic parameters such as pleomorphism, necrosis, and vascular invasion are poor prognostic parameters. It is found logical to combine these parameters in a scoring system, in a way similar to scoring adrenocortical tumors (Thompson et al., 2020; Kimura et al., 2018). Subsequently, a system is developed using six criteria (histologic pattern, cellularity, coagulation necrosis, vascular/capsular invasion, MIB-1 immunoreactivity, and produced catecholamine types) and tumors are classified as well, moderately, and poorly differentiated (with 10-year survival rates of 83%, 38%, and 0%, respectively). In one series, 84% of the tumors that invaded blood vessels and all cases associated with metastases had a tetraploid or aneuploid pattern and another unfavorable prognostic factors was high proliferative index (as measured with MIB-1 or topoisomerase alpha II staining (Elder et al., 2003; Lupşan et al., 2016; Vyakaranam et al., 2019; Parenti et al., 2012). Reported proportions of malignant PGL vary considerably between most genotype-phenotype studies, ranging from 31 to 71.4% in *SDHB*-mutation carriers (van Hulsteijn, 2012; Huang et al., 2018).

In our series, there was a statistically significant difference between tumor type and malignancy potantial according to PASS classification. The lowest potential was found in HNPGLs (15.07%) and the highest in Pheo (51.85%). However, we know that histopathological features are not reliable and the only dependable malignancy criteria of PPGL tumors is metastasis. There were four metastatic cases in our series. One of the cases with metastases was located at right carotid region and had multiple metastates to lung, sacrum and brain. One of the patients located at left adrenal had metastasized to lymph node and the other to the rib. The patient with a retroperitoneal mass had a liver metastasis. Only one patient with metastasis showed loss of SDHB expression. The common features of all metastatic cases were malignancy potential according to PASS classification (> 3 points), pleomorphism and vascular invasion. It seems likely that histopathological features are more predictive for malignancy than *SDHB* mutation.

HNPGLs are a group of neurogenic tumors with distinct clinical features. Its incidence is low (1/ 30 thousand – 1/ 100 thousand); it accounts for 0.6% of head and neck tumors and 15% of the cases are malignant. It usually does not have endocrine function. It is most commonly found in the carotid body (about 60% of HNPGLs). It can also occur in the jugular foramen, the tympanum, or the vagus nerve. HNPGL might be related to genetic abnormalities and environmental factors; can be familial or sporadic. Familial tumors are generally inherited in autosomal dominant pattern and, 80% are multiple. Multiple PGL occur as sporadic occurrences in only 10% to 20% of patients. Baysal et al., (2000), ﬁrst discovered the mutation of the *SDHD* gene in patients with HNPGL (Ding et al., 2019). In our series, HNPLGs accounted for 64.03 % of the cases. Most of these cases were located close to carotid artery. Of the cases, 4.11% were younger than 31 years and 15.07% had a malignancy potential according to PASS classification.* SDHB* mutation (16.44%) was less frequent than TAPPGLs and more than Pheos. We observed a multiple tumor in a 42 years old woman without a *SDHB* mutation. As a result, HNPLGs are the most frequent cases and found in older patients. Their histopathological malignancy potential according to PASS classification is statictically significanly lower than the other two groups (p<0.0001).

It is well known that 30–50% of Pheos and PGLs develop due to underlying germline mutations. Any patient with a PGL or Pheo, particularly in case the following findings are present, should be evaluated for a hereditary PPGL syndrome (Lenders et al., 2014): If the tumors are multiple (i.e., >1 PGL or Pheo), including bilateral adrenal Pheo, multifocal with multiple synchronous or metachronous tumors, recurrent, have early onset (i.e., age <45 years), extra-adrenal, metastatic with a family history of PGL or Pheo, if there are relatives with unexplained or incompletely explained sudden death. However, many individuals with hereditary PPGL syndrome might present with a solitary tumor of the skull base or neck, thorax, abdomen, adrenal, or pelvis and no family history of PGL or Pheo (Else et al., 2008). 

Mutations involved in the pathogenesis of PPGL have been recently classified into 4 categories; 

1. Pseudohypoxemia group involving mainly the SDH subgroup (SDHA, SDHB, SDHC, SDHD and SDHAF2), fumaratehydratase (FH) and the VHLEPAS1 subgroup; 

2. The Tyrosine kinase group (RET, NF1, MAX, TMEM127 and HRAS); 

3. WNT-related pathway (somatic mutations in MAML3 and CSDE1); and 

4. Adrenocortical admixture group. 

Most mutations involve the pseudohypoxemia group with the vast majority occurring in SDHB and to a lesser extent in SDHC and SDHD. These mutations occur commonly in PGL, but very rarely in Pheo (Crona et al., 2017; Flynn et al., 2015; Albattal et al., 2019). *SDH* mutations are commonly observed in a number of hereditary and sporadic malignancies. Mutations in any of the four SDH subunits lead to the disintegration of the SDH complex and result in a complete loss of SDH enzymatic activity (Oudijk et al., 2019).

Patients with* SDHB* mutations are younger, and more commonly have extra-adrenal tumors (Nazar et al., 2019). We also observed *SDHB* mutation the younger age group more frequently. *SDHB* mutation were seen in 4 cases (40%) at age ≤ 30 vs. 16 cases (15.4 %) at age> 31.

*SDHB* expression of PPGL is detected in a number of articles. The results are inconsistent and frequency of *SDHB* mutation ranged between 6.7% and 63.0% (Albattal et al., 2019; Fishbein et al., 2017; Pandit et al., 2016). We detected a similar frequency of mutation like Albattal (2019) and found 20 *SDHB* mutations in our 114 randomly selected PPGL series (17.54%). 

Frequency of *SDHB* mutation in different anatomic locations of PPGLs is a matter of interest. Generally* SDHB* mutation was detected most frequently in TAPPGLs and rarely in PCCs (Pandit et al., 2016). In a non-syndromic Pheo series from Europe, *SDHB* mutations were found in 12 of 271 (4.4%) (Neumann et al., 2002). In another study,* SDHB* mutation was detected most frequently in TAPPGLs. Immunohistochemistry is recommended to guide genetic screening especially in abdominal PGLs (Currás-Freixes et al., 2015). In Albattal’s study (2019), the majority of cases with* SDHB* mutations presented with abdominal PGL (14/21 cases, 66.7%) (Albattal et al., 2019). Five cases presented with HNPGL (13,5%).Two out of 32 (6.3%) Pheo cases carried* SDHB* mutations. We detected 3/27 (11.11%) *SDHB* mutations in Pheos. One of the cases was with RCC and the other was with retroperitoneal PGL. In one case renal cell carcinoma was observed in right kidney. The other had retroperitoneal composite PGL Third case had rib bone metastasis. 

We observed* SDHB* mutation in 17.54% of all tumors. The most frequent mutation was detected in TAPPGLs (35-71 %) followed by HNPGLs (16.44%) and Pheos (11-12% ) (p<0.03).

There is a consensus about the relation between SDHB mutation and unfavourable prognosis. Germline pathogenic variants in SDHB are generally associated with higher morbidity and mortality than pathogenic variants in the other *SDHx* genes (Ricketts et al., 2010; Andrews et al., 2018). Especially SDHB-mutation carriers have higher risk of developing a metastatic disease and shorter survival than patients with a malignant PPGL, but without SDHB mutations (Benn et al., 2015; Nazar et al., 2019; Gimenez-Roqueplo et al., 2003; King et al., 2011; Brouwers et al., 2006; Turkova et al., 2016; Hamidi et al., 2017).

*SDHB* mutations are strongly associated with extra-adrenal sympathetic paragangliomas with an increased risk of metastatic disease, and less frequently, with Pheos and parasympathetic PGLs (Andrews et al., 2018). Up to 50% of persons with metastatic extra-adrenal paragangliomas have a germline SDHB pathogenic variant (Else et al., 2008; Fishbein et al., 2017). SDHB mutations are especially related to sporadic malignant Pheo with poor prognosis, and up to 40% of patients with metastatic disease harbor mutations in this gene (Andrade, 2018). In our study we observed the highest frequency of SDHB mutation in TAPPGLs (35.71%) and lowest frequency in Pheos (2 case 11.12%). Both of Pheo cases seems likely syndromic; one case was a 24 years old male with RCC and the other was a 62 years old male with another paraganglioma focus at retroperitoneum. The only metastatic case with SDHB mutation was a HNPGL. 

Loss of the SDH complex is described in extra-adrenal paragangliomas, gastrointestinal stromal tumors, renal cell carcinomas and rarely in other epithelial tumors (Nazar et al, 2019). In addition to PPGLs, other malignant tumors may be seen in cases with SDHB mutation. The most common tumor is RCC. Andrews et al., (2018) reported that 15/751 (2.56%) SDHD carriers had a renal tumour. Other rare tumours found in SDHB carriers are thyroid tumors, pituitary adenoma, parathyroid adenoma and pulmonary carcinoid tumour. In our series, we had 2 cases with RCC, one of which was with *SDHB* mutation. 

*SDHB* germ line mutation is the most common genetic disorder in PPGLs. Its detection with immunohistochemistry is a practical, reliable, and unexpensive method to determine the functionality of genetic variants of various *SDHB* mutations and to guide genetic screening. (Oudijk et al, 2019; Currás-Freixes et al., 2015; Papathomas et al., 2015). Normally, SDHB staining is positive in all cells, with strong granular and cytoplasmic labeling representing mitochondrial localization of the protein (Oudijk et al., 2019; van Nederveen et al. 2009]. Non-tumoral cells such as endothelial cells, fibroblasts, or lymphocytes can be used as an internal positive control. If any of the subunits of the SDH complex is lost due to (epi) mutation, the entire complex becomes unstable and the SDHB subunit is degraded in the cytoplasm (Oudijk et al., 2019; Gill , 2012). This loss of the SDHB protein can be shown by SDHB immunohistochemistry. As a result, tumors with inactivating *SDHA, SDHB, SDHC, SDHD*, or *SDHAF2* mutations demonstrate loss of cytoplasmic SDHB staining. If SDHB staining in the tumor cells is evidently less intense compared to non-tumoral cells, or if it shows a weak diffuse cytoplasmic blush instead of a granular staining pattern, SDHB immunohistochemistry should be regarded negative (Oudijk et al, 2019; Elder et al., 2003). In our cases, we observed complete loss of SDHB staining in mutated tumor cells. 

 SDHB immunostaining might appear falsely negative in tumor areas with clear cytoplasm, for example in clear cell RCC. In such cases, the best approach is to look for areas with eosinophilic cytoplasm for interpretation of the staining. In a multicenter study, SDHB immunohistochemistry was shown to be a reliable technique with almost 90% consensus in a group of seven reviewers. The positive and negative predictive value of the seven reviewers of SDHB immunohistochemistry to detect *SDH* mutations ranged from 67 to 93%, and from 90 to 99%, respectively (Oudijk et al, 2019; Papathomas et al, 2015).

Some tumors show a weak diffuse cytoplasmic SDHB immunostain, particularly PPGL with *SDHD* mutations [Oudijk et al., 2019; Gill, 2012]. Even though SDHD immunohistochemistry cannot be used to specifically detect SDHD-mutated tumors, the staining provides a complement to difficult to interpret SDHB immunostainings. Diffuse or discordant negative cytoplasmic SDHB staining has also been described in a minor subset of VHL-and NF1 mutated PCC/PGL. Therefore, if SDH genetic testing of an SDHB-immunonegative tumor does not show a mutation, *SDHC* promoter methylation, VHL, and/orNF1 molecular testing is recommendable (Oudijk et al., 2019; Papathomas et al., 2015). In our cases, we observed complete loss of SDHB staining in mutated tumor cells.

Generally the biological behaviour and malignancy potential of some endocrine tumors are unpredictable and some proliferation markes are used as a predictor of biological behaviour. The Ki-67 nuclear antigen is a nuclear protein that is abundantly expressed in G1 through S phase of the cell cycle but is rapidly degraded after mitosis. Because the monoclonal antibody MIB-1 recognizes a formalin-resistant epitope of Ki-67, it can be used in routinely fixed, paraffin-embedded tissue.Ki-67/MIB-1 immunostaining has become clinically relevant for other endocrine tumor types in situations where discrimination between benign and malignant tumors is challenging. For example, in the investigation of pituitary tumors and their potential aggressive/ invasive behavior, a cutoff level of proliferative activity has even been adapted in World Health Organization classification of endocrine tumors (Elder et al., 2003).

There are a number of studies on Ki-67 immunoreactivity of PPGLs. Some papers claim that Ki-67 proliferation index can predict malignancy in this tumor group (Lupşan et al., 2016; Elder et al., 2003). Different Ki-67 index levels between >3% and >5% are proposed (Lupşan et al., 2016; Parenti et al., 2012). 

Lupsan et al., (2016) claimed that a Ki-67 index >3% could predict the malignant potential, since benign PCCs have never been shown to have scores >3. PASS and specifically Ki-67 mitotic index have a powerful impact on the survival rate and could be considered as possible predictors of malignancy (Parenti et al., 2012; Elder et al., 2003). Ki-67 antigen is very sensitive to fixation and prolonged storage, which could explain failure of detecting elevated Ki-67/MIB-1 expression levels in some malignant tumors. The combined use of Ki-67/MIB-1 and hTERT may become a valuable diagnostic addition for Pheo and abdominal PGLs (Elder et al., 2003). Ocal (2014) did not observe a relation between Ki-67 index and but found a statistically significant correlation between Ki-67 proliferation index and capsule invasion (Ocal et al., 2014). In our study we could not find a statistically significant difference between Ki-67 index and malignancy potential. In PGL cases under 30 years old with an Ki67 index>3 had a 5X increase in malignancy potential. However sample size of group was small and statistically insignificant. Besides, in cases with Ki67 index >3, loss of SDHB expression showed a prominent increase in Pheo cases over 30 years old (9X). There is a statistically significant difference between anatomical location and Ki-67 index. Tumors with Ki-67 index >3 are more frequent in TAPPGLs (50.00%) and in contrast, rare in Pheos (11.12%)

In conclusion, high percent of PPGL tumors are syndromic; most of cases with germ- line mutations are solitary and without a family history. Immunohistochemical negativity of SDHB is not only an indicator of SDHB, but of all *SDHx* mutations that consist most of the germ line mutations and sometimes of VHL-and NF1. We detected immunohistochemical negative staining of in 17.54% of all tumors and more than one third of TAPPGLs.

Routine application of SDHB immunohistochemistry to the PPGL tumors is a practical and unexpensive method that can be used to select the candidate patients for molecular pathological examination especially in TAPPGLs. This may help to identify the syndromic cases and their families who are in a risk of having secondary malignancies. 

TAPPGLs, HNPGLs and Pheos are the tumors with similar origin and sharing similar histopathological properties, but their SDHB expression (p<0.02), malignancy potential according to PASS classification (p<0.0001), Ki-67 proliferation index (p<0.0001) are statistically different from each other. Therefore, follow up protocols as well as clinical and therapeutical approach must be different in these subgroups. 

## Author Contribution Statement

AEG,SHK, NOB contributed to the design and implementation of the research; AEG, SHK, NOB and YSH to the analysis of the results; SS, YSG, AEG to the writing of the manuscript and AEG, SHK, NOB, YSG, SS and NE to the revision of the draft and completion of the manuscript.
